# Identification of Common Oncogenic Genes and Pathways Both in Osteosarcoma and Ewing's Sarcoma Using Bioinformatics Analysis

**DOI:** 10.1155/2022/3655908

**Published:** 2022-05-05

**Authors:** Jingwei Zhang, Junchao Huang, Wenjun Liu, Liang Ding, Dongdong Cheng, Haijun Xiao

**Affiliations:** ^1^Department of Orthopedics, Shanghai Fengxian District Central Hospital/Shanghai University of Medicine & Health Sciences Affiliated Sixth People's Hospital South Campus, Shanghai 201499, China; ^2^Department of Orthopedics, Shanghai Fengxian District Central Hospital/Anhui University of Science and Technology Affiliated Fengxian Hospital, Shanghai 201499, China; ^3^Department of Orthopedics, Shanghai Sixth People's Hospital, Shanghai 200023, China

## Abstract

This study was aimed at exploring common oncogenic genes and pathways both in osteosarcoma and Ewing's sarcoma. Microarray data were obtained from the Gene Expression Omnibus (GEO) database. Differentially expressed genes (DEGs) were identified using the limma package. Then, protein-protein interaction (PPI) networks were constructed and hub genes were identified. Furthermore, functional enrichment analysis was analyzed. The expression of common oncogenic genes was validated in 38 osteosarcoma and 17 Ewing's sarcoma tissues by RT-qPCR and western blot compared to normal tissues. 201 genes were differentially expressed. There were 121 nodes and 232 edges of the PPI network. Among 12 hub genes, hub genes FN1, COL1A1, and COL1A2 may involve in the development of osteosarcoma and Ewing's sarcoma. And they were reduced to expression both in osteosarcoma and Ewing's sarcoma tissues at mRNA and protein levels compared to normal tissues. Knockdown of FN1, COL1A1, and COL1A2 enhanced the cell proliferation and migration of U2OS under the restriction of cisplatin. Our findings revealed the common oncogenic genes such as FN1, COL1A1, and COL1A2, which may act as antioncogene by enhancing cisplatin sensitivity in osteosarcoma cells, and pathways were both in osteosarcoma and Ewing's sarcoma.

## 1. Introduction

Osteosarcoma and Ewing's sarcoma are the two most common primary bone malignancies in children and adolescents [1, 2]. Although therapeutic strategies have made enormous progress, patients' survival time has only improved marginally [3, 4]. The high morbidity and mortality of these two diseases require more research to characterize and understand their potential molecular mechanisms [5, 6]. More in-depth study of molecular pathways affected by these two diseases will facilitate better development of therapy. Increasing evidence suggests that many cellular signaling molecules are involved in tumorigenesis, and many specific therapeutic targets have been identified based on them [7, 8]. Furthermore, at molecular levels, these two diseases have many genetic features in common. Researches on the molecular pathogenesis of osteosarcoma and Ewing's sarcoma are still limited, whereas better diagnostic and prognostic tools are still lacking.

To analyze the pathogenesis of osteosarcoma and Ewing's sarcoma, multiple DEGs have been identified between cancer tissues and normal samples among the population using multiple methods [9, 10]. Because of the large individual differences and heterogeneity of these two diseases, DEGs identified may not accurately describe a single disease sample [11–13]. Moreover, it is sometimes difficult to compare gene expression measurements from different samples and platforms [14]. It has been suggested direct comparisons of gene expression levels between diseased tissues and adjacent normal tissues [14]. However, it lacks proper statistical control, and its applications are limited because corresponding normal tissue specimens are usually not available. Therefore, unlike the previous studies, this study was aimed at identifying key genes and pathways common in osteosarcoma and Ewing's sarcoma utilizing integrated bioinformatics methods.

## 2. Results

### 2.1. Identification of DEGs and Their Biological Functions for Osteosarcoma

**Figure 1:**
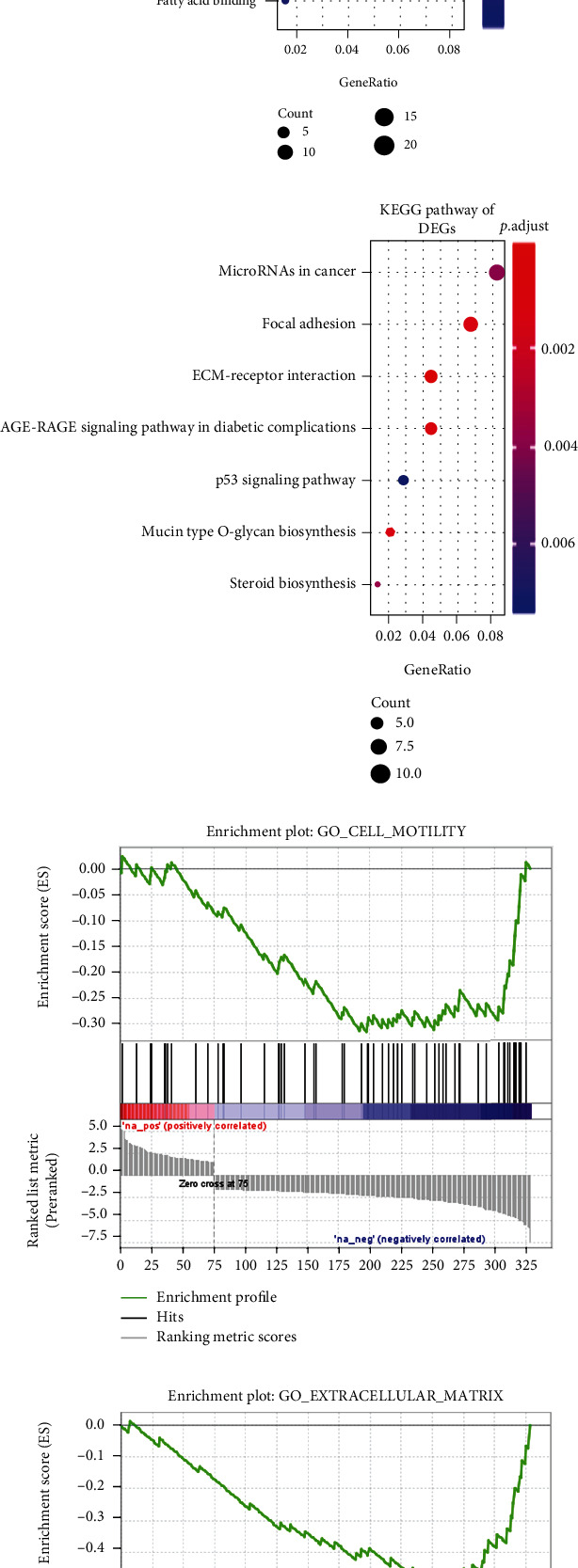
Identification of DEGs and their biological functions for osteosarcoma. (a) Heatmap showing 329 DEGs between osteosarcoma cells and mesenchymal stem cells. Red represents upregulated genes and green represents downregulated genes. GO including (b) biological process, (c) cell component, and (d) molecular function and (e) KEGG enrichment results depicting underlying biological functions for these DEGs. (f–i) GSEA results based on these DEGs, including cell motility, extracellular matrix, signaling receptor binding, and regulation of cell adhesion.

Using the limma package, DEGs with ∣logFC | >1.5 and *p* < 0.05 were screened between 5 cases of osteosarcoma cells and 1 case of mesenchymal stem cell from the GSE70414 dataset. As a result, a total of 329 DEGs were identified for osteosarcoma, including 75 upregulated and 254 downregulated genes. The expression patterns of these genes between mesenchymal stem and osteosarcoma cells are shown in Figure 1(a). Supplementary Table 1 lists the top ten upregulated genes for osteosarcoma cells, such as LOC728613, ZIC2, and CD24. Furthermore, the highest ten downregulated genes for osteosarcoma are listed in Supplementary Table 2.

To explore potential biological functions and pathways of the 329 DEGs in osteosarcoma cells, functional enrichment analysis was performed utilizing the clusterProfiler package. GO terms included biological process (Figure 1(b)), cell component (Figure 1(c)), and molecular function (Figure 1(d)). KEGG results showed that these genes were mainly enriched in several pathways related to cancer, such as microRNAs in cancer and p53 signaling pathway (Figure 1(e)). Detailed information about KEGG pathway is listed in Supplementary Table 3. Based on these osteosarcoma-related DEGs, GSEA was also presented. Our data suggested that cancer metastasis-related biological processes were significantly enriched, such as cell motility (Figure 1(f)), extracellular matrix (Figure 1(g)), signaling receptor binding (Figure 1(h)), and regulation of cell adhesion (Figure 1(i)).

**Figure 2:**
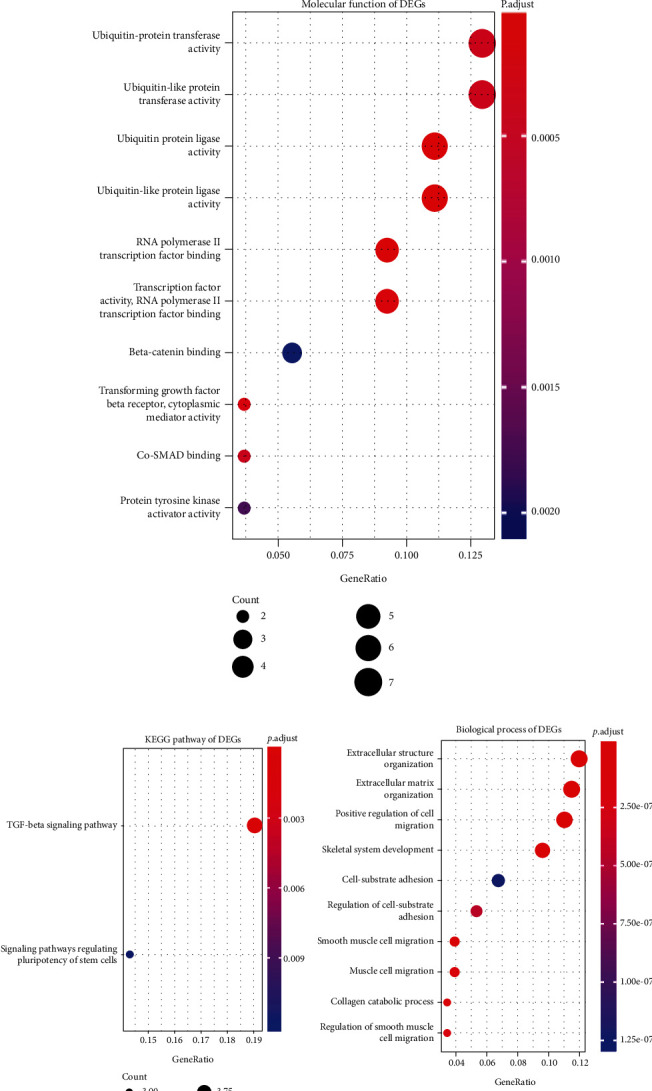
Functional enrichment analysis results of up- and downregulated genes for osteosarcoma. (a) Biological processes; (b) cell component; (c) molecular function; and (d) KEGG for up-regulated genes. (e) Biological processes; (f) cell component; (g) molecular function, and (h) KEGG for downregulated genes.

We further probed into the biological functions of up- and downregulated genes, respectively. The data showed that upregulated genes were distinctly involved in regulation of binding (Figure 2(a)), anchored component of external side of plasma membrane (Figure 2(b)), ubiquitin (Figure 2(c)), and TGF-*β* signaling pathway (Figure 2(d)). Downregulated genes could significantly participate in regulating extracellular structure (Figure 2(e)), extracellular matrix (Figure 2(f)), cell adhesion molecule binding (Figure 2(g)), and focal adhesion (Figure 2(h)).

### 2.2. Identification of DEGs and Their Biological Functions for Ewing's Sarcoma

**Figure 3:**
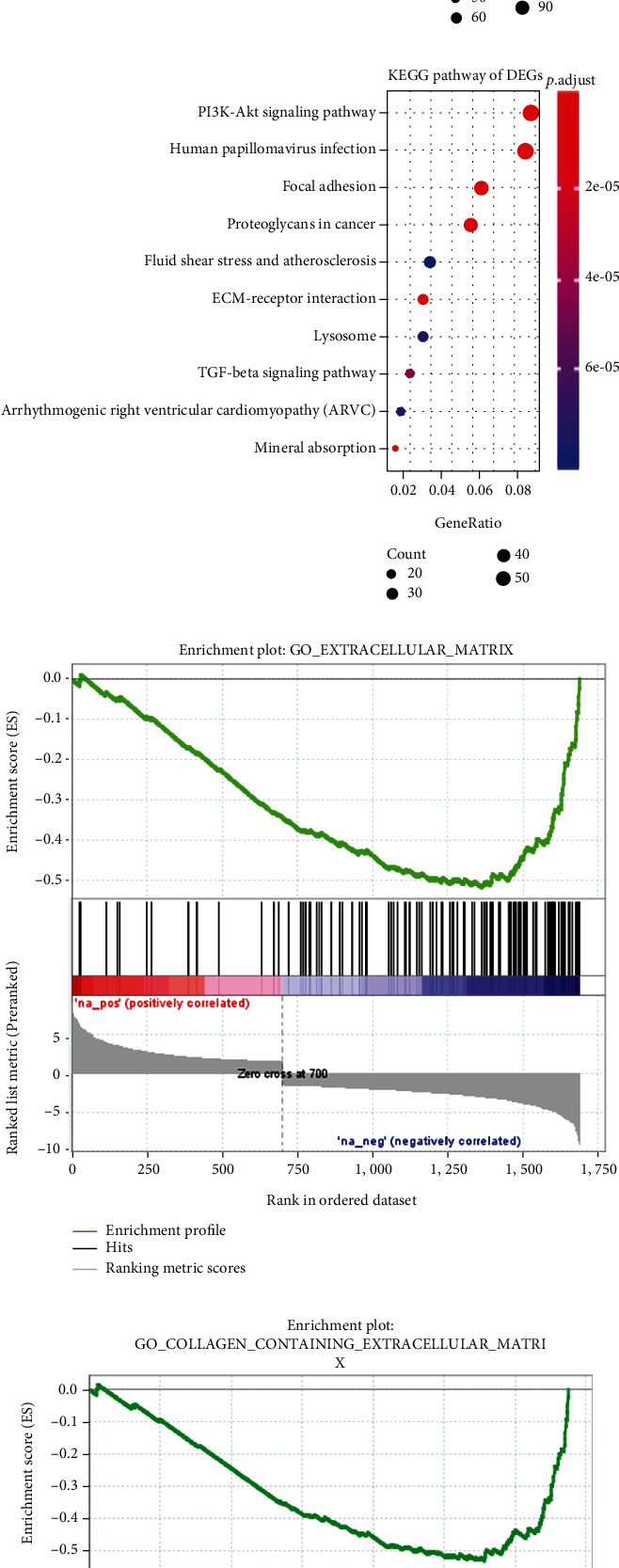
Identification of DEGs and their biological functions for Ewing's sarcoma. (a) Heatmap showing 1688 DEGs between Ewing's sarcoma and mesenchymal stem cells. Red represents upregulated genes and green represents downregulated genes. GO including (b) cell component, (c) molecular function, and (d) biological process and (e) KEGG enrichment results depicting underlying biological functions for these DEGs. (f–i) GSEA results according to these DEGs, including extracellular matrix, collagen-containing extracellular matrix, DNA binding transcription factor activity, and cell substrate adhesion.

We further analyzed DEGs between 5 cases of Ewing's sarcoma cells and 1 case of mesenchymal stem cell from the GSE70826 dataset. A total of 1688 DEGs with ∣logFC | >1.5 and *p* < 0.05 were identified between Ewing's sarcoma cells and mesenchymal stem cells, composed of 700 upregulated and 988 downregulated genes (Figure 3(a)). The top ten upregulated and downregulated genes are listed in Supplementary Table 4 and Supplementary Table 5, respectively. The potential functions and pathways of DEGs for Ewing's sarcoma cells were explored using the clusterProfiler package. The GO results showed that these genes were significantly related to the extracellular matrix (Figure 3(b)), cell adhesion molecular binding (Figure 3(c)), and extracellular structure organization (Figure 3(d)). Furthermore, these DEGs were mainly involved in several KEGG pathways associated with cancer, like PI3K-Akt signaling pathway and TGF-*β* signaling pathway (Figure 3(e)). The genes in the KEGG pathways are listed in Supplementary Table 6. GSEA results also demonstrated the extracellular matrix (Figure 3(f)), collagen-containing extracellular matrix (Figure 3(g)), DNA binding transcription factor activity (Figure 3(h)), and cell substrate adhesion (Figure 3(i)).

**Figure 4:**
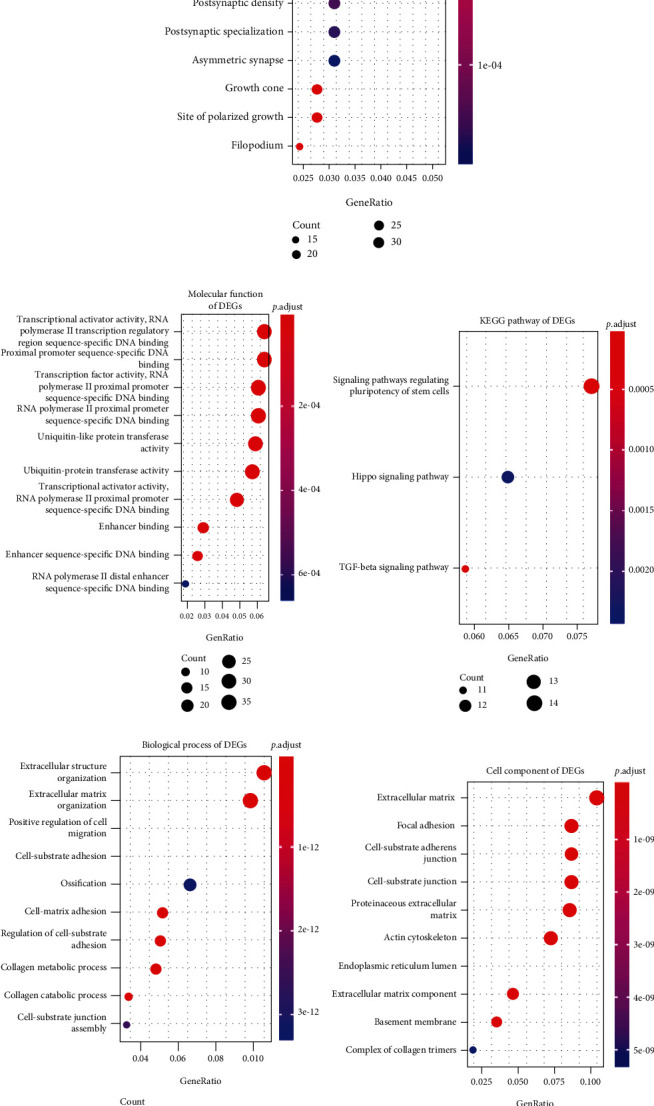
Functional enrichment analysis results of up- and downregulated genes for Ewing's sarcoma. (a) Biological processes; (b) cell component; (c) molecular function; and (d) KEGG for upregulated genes. (e) Biological processes; (f) cell component; (g) molecular function and (h) KEGG for down-regulated genes.

The biological functions of up- and downregulated genes were separately analyzed for Ewing's sarcoma. Our data suggested that upregulated genes were mainly enriched in axon development (Figure 4(a)), neuronal cell body (Figure 4(b)), transcription (Figure 4(c)), and signaling pathways regulating pluripotency stem cells (Figure 4(d)). Meanwhile, downregulated genes exhibited a significant correlation with extracellular structure organization (Figure 4(e)), extracellular matrix (Figure 4(f)), cell adhesion molecular binding (Figure 4(g)), and PI3K-Akt signaling pathway (Figure 4(h)).

### 2.3. Common DEGs Both in Osteosarcoma and Ewing's Sarcoma Compared to Mesenchymal Stem Cells

We comprehensively analyzed the common DEGs between osteosarcoma and Ewing's sarcoma. A total of 201 genes were differentially expressed both in osteosarcoma and Ewing's sarcoma compared to mesenchymal stem cells (Supplementary Figure 1A). Heatmap depicted the difference in the expression pattern of these common DEGs between osteosarcoma cells (Supplementary Figure 1B) and Ewing's sarcoma cells (Supplementary Figure 1C) compared to mesenchymal stem cell, respectively.

### 2.4. PPI Networks of Common DEGs Both in Osteosarcoma and Ewing's Sarcoma

To explore relationships between these common DEGs, the 201 DEGs were analyzed by the STRING. Then, a PPI network was constructed and visualized using the Cytoscape. There were 121 nodes and 232 edges of the PPI network (Supplementary Figure 2A). Genes with degree > 10 were considered as hub genes (Supplementary Table 7). In addition, a subnetwork was then constructed using the Cytoscape MCODE, composed of 12 nodes and 51 edges (Supplementary Figure 2B). The hub genes in the network were all downregulated both in osteosarcoma and Ewing's sarcoma cells compared to mesenchymal stem cells.

### 2.5. Functional Enrichment Analysis of Common DEGs Both in Osteosarcoma and Ewing's Sarcoma

The Cytoscape plugin ClueGO was used to visualize the functional enrichment analysis results of common DEGs both in osteosarcoma and Ewing's sarcoma compared to mesenchymal stem cells. Biological processes enriched by these genes were shown in Supplementary Figure 3, such as sprouting angiogenesis and regulation of cell-substrate adhesion. Cell component results showed that these genes were mainly enriched in collagen-containing extracellular matrix, extracellular matrix, extracellular matrix component, platelet alpha granule lumen, and so on (Supplementary Figure 4). As for molecular function, these genes were significantly associated with protease binding, collagen binding, heparin binding, integrin binding, and metalloendopeptidase activity (Supplementary Figure 5). KEGG pathway enrichment analysis results showed that these genes were mainly involved in ECM-receptor interaction (including FN1, COL1A1, COL1A2, COMP, ITGA5, and THBS1) and AGE-RAGE signaling pathway in diabetic complications (including FN1, COL1A1, COL1A2, SERPINE1, and AGTR1) in Supplementary Figure 6.

### 2.6. Validation of Common Oncogenic Genes in Osteosarcoma and Ewing's Sarcoma Tissues

**Figure 5:**
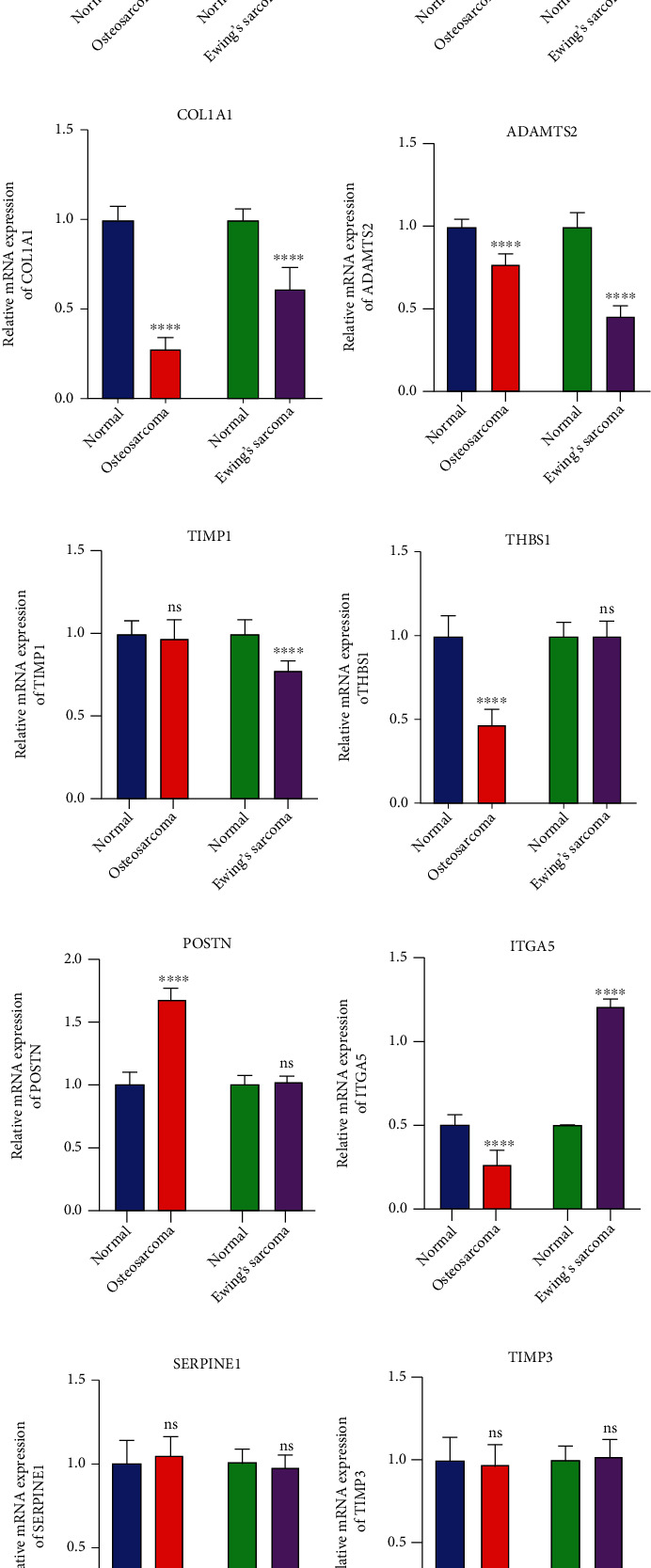
Validation of common oncogenic genes in osteosarcoma and Ewing's sarcoma tissues. RT-qPCR results detecting the mRNA expression of (a) FN1, (b) COL1A2, (c) COL1A1, (d) ADAMTS2, (e) TIMP1, (f) THBS1, (g) POSTN, and (h) ITGA5, (i) SERPINE1, and (j) TIMP3 between osteosarcoma or Ewing's sarcoma tissues and normal tissues. (k–n) Western blot showing the expression of FN1, COL1A2, and COL1A1 proteins between osteosarcoma or Ewing's sarcoma tissues and normal tissues. Ns: not significant; ∗∗∗∗*p* < 0.0001.

Common oncogenic genes were further validated in 38 osteosarcoma and 17 Ewing's sarcoma tissues by RT-qPCR and western blot compared to adjacent normal tissues. Our RT-qPCR results showed that FN1 (Figure 5(a)), COL1A2 (Figure 5(b)), COL1A1 (Figure 5(c)), and ADAMTS2 (Figure 5(d)) were downregulated both in osteosarcoma and Ewing's sarcoma tissues compared to normal tissues. There was no significant differences in TIMP1 expression between osteosarcoma and normal tissues. But TIMP1 was reduced expression in Ewing's sarcoma tissues than normal tissues (Figure 5(e)). In Figure 5(f), THBS1 had a lower expression in osteosarcoma than normal tissues. No significant difference was detected between Ewing's sarcoma tissues and normal tissues. Furthermore, POSTN exhibited a significantly higher expression in osteosarcoma than normal tissues. Meanwhile, in Ewing's sarcoma, tissues are not (Figure 5(g)). ITGA5 was markedly downregulated in osteosarcoma tissues but upregulated in Ewing's sarcoma tissues compared to normal tissues (Figure 5(h)). However, no significant difference was noted in SERPINE1 and TIMP3 expression between osteosarcoma or Ewing's sarcoma tissues and normal tissues (Figures 5(i) and 5(j)).

### 2.7. Knockdown of FN1, COL1A1, and COL1A2 Enhanced the Cell Proliferation and Migration of U2OS under the Restriction of Cisplatin

**Figure 6:**
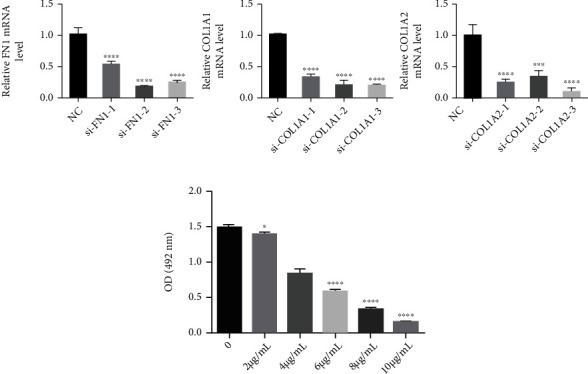
(a) The effectiveness of si-FN1, Si-COL1A1, and Si-COL1A2 knockdown plasmids was verified by PCR experiment. (b) The concentration of cisplatin for in vitro administration was determined by CCK-8 assay. ∗*p* < 0.05, ∗∗∗*p* < 0.001, and ∗∗∗∗*p* < 0.0001.

**Figure 7:**
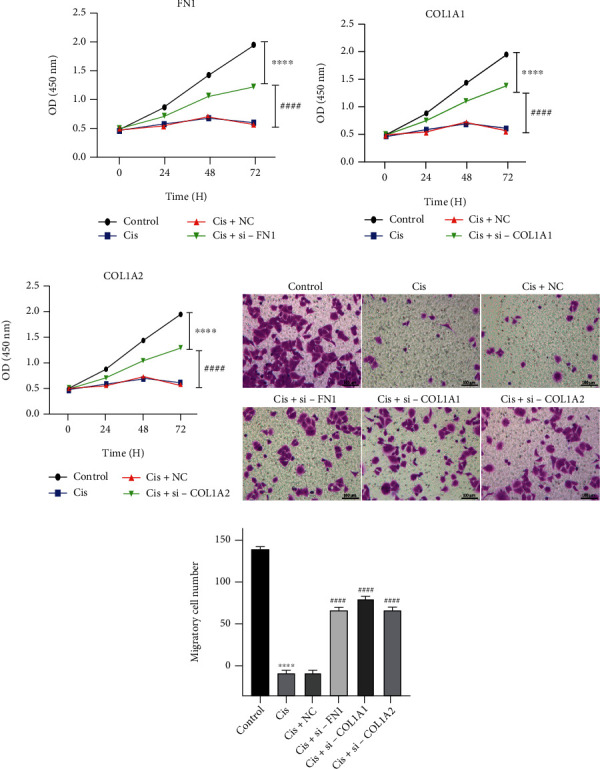
FN1, COL1A2, and COL1A1 enhance cisplatin sensitivity in osteosarcoma cells. (a–c) The effects of FN1, COL1A2, or COL1A1 knockdown on cell viability of U2OS cells were measured by CCK-8 assay (*N* = 3). (d and e) The migration capacity of FN1, COL1A2, or COL1A1 knockdown U2OS cells treated with cisplatin was analyzed by transwell assays. ∗∗∗∗*p* < 0.001 compared with the control group and ####*p* < 0.001 compared with the Cis+NC group.

**Figure 8:**
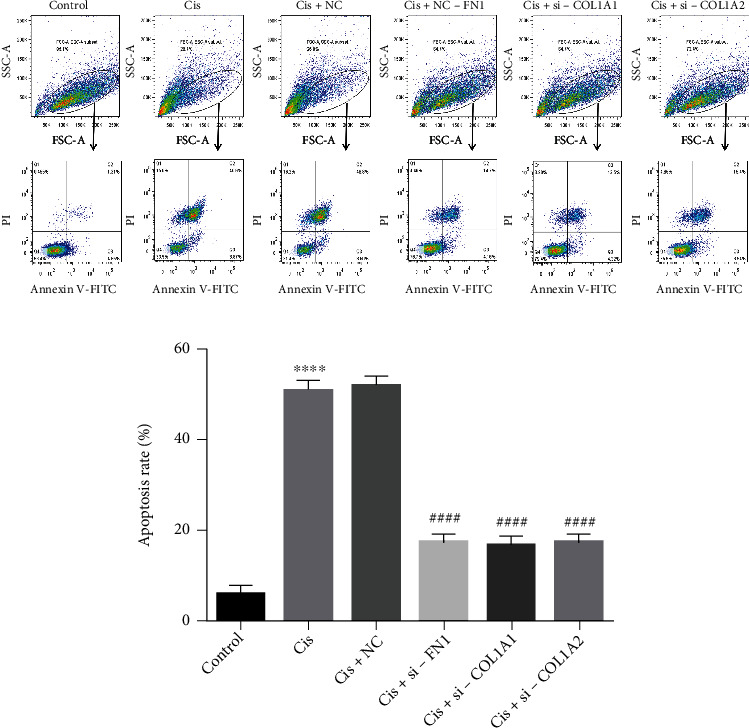
Flow cytometric analysis was used to detect the effects of FN1, COL1A2, or COL1A1 knockdown combined with cisplatin and cisplatin alone on cell apoptosis of U2OS cells. ∗∗∗∗*p* < 0.001 compared with the control group, ####*p* < 0.001 compared with the Cis+NC group.

According to our results (Figure 5), the expression of FN1, COL1A1, and COL1A2 were downregulated in osteosarcoma and Ewing's sarcoma, which often act as oncogenes, suggesting that they were more likely to play roles of antioncogene in osteosarcoma and Ewing's sarcoma. In order to verify their potential effects of antioncogene, si-FN1, si-COL1A1, si-COL1A2, and si-NC were transferred into U2OS cells after the effectiveness of the knockout plasmids has been verified (Figure 6(a)). Cisplatin was used to limit the cellular phenotype of U2OS in vitro, which currently was the most commonly used chemotherapeutic drug in clinical treatment of osteosarcoma; the concentration of cisplatin for in vitro administration was determined by CCK-8 assay (Figure 6(b)). The cisplatin half maximal inhibitory concentration (IC50) of U2OS cells was 4.7 *μ*g/ml calculated by SAS software (USA). Then, transfected U2OS cells were incubated with 4.7 *μ*g/ml cisplatin for subsequent analysis. The results showed that knockdown of FN1, COL1A1, or COL1A2 enhanced their resistance to apoptosis and cell reproductive capacity under the restriction of cisplatin, as indicated in the cell proliferation rate (####*p* < 0.001) (Figures 7(a)–7(c)), and a decrease in the number of Annexin V-PI positive cells (####*p* < 0.001) (Figure 8), compared with si-NC control treated cells. In addition, transwell results showed that the migration ability of cisplatin restricted U2OS cells was restored after knockdown of these antioncogenes, which meant that the existence of FN1, COL1A1, or COL1A2 may inhibit the U2OS cell migration ability (Figures 7(d) and 7(e)). These results indicated that FN1, COL1A1, or COL1A2, which may act as oncogene by enhancing cisplatin sensitivity, might be involved in the progression and development of osteosarcoma.

## 3. Discussion

The cell line panel provides a valuable model system for analyzing gene expression in osteosarcoma and Ewing's sarcoma. In this study, a comprehensive bioinformatics approach was used to analyze gene expression in osteosarcoma and Ewing's sarcoma cell lines compared to normal controls. We identified 329 DEGs with ∣logFC | >1.5 and *p* < 0.05 in 5 cases of osteosarcoma cells compared to 1 case of mesenchymal stem cell using the limma package, including 75 upregulated and 254 downregulated genes. Among 75 upregulated genes, the top ten genes according to fold change included LOC728613, ZIC2, CD24, ABLIM1, MYLIP, SIPA1L2, RHPN2, S100A4, LHX2, and LOC100996740. Furthermore, the top ten downregulated genes included FBLN5, PLD5, TRIM22, FN1, KCTD12, CTSK, ABI3BP, HAS2, POSTN, and TACSTD2. Functional enrichment analysis results revealed that these DEGs were involved in several pathways related to cancer, such as microRNAs in cancer and p53 signaling pathway. We concluded that BCL2L11, E2F2, FOXP1, HMOX1, ITGA5, MIR34A, MARCKS, ZEB1, THBS1, TIMP3, VIM, and RPS6KA5 were enriched in the microRNAs in cancer pathway. It has been recognized that miRNAs, a class of small noncoding RNA, are involved in tumorigenesis and development of various cancers including osteosarcoma by regulating protein expression at the posttranscriptional level [15‒18]. Furthermore, DDB2, IGFBP3, SERPINE1, THBS1, and TP53I3 were enriched in p53 signaling pathway. Increasing evidence suggests that abnormal expression of many genes could activate p53 signaling pathway in osteosarcoma [19, 20]. Thus, the above genes might be involved in the development of osteosarcoma, which require further experimental validation.

Similarly, we analyzed DEGs in 5 cases of Ewing's sarcoma cells compared to 1 case of mesenchymal stem cell. A total of 1688 DEGs were identified, including 700 up- and 988 downregulated genes. The top ten upregulated genes were as follows: COL6A3, COL8A1, CTHRC1, SRGN, TGFBI, MICAL2, ITGBL1, HAS2, LGALS3, and SERPINE1. Downregulated genes included COL6A3, COL8A1, CTHRC1, SRGN, TGFBI, MICAL2, ITGBL1, HAS2, LGALS3, and SERPINE1. Functional enrichment analysis results showed that these DEGs were mainly involved in several KEGG pathways associated with cancer, like PI3K-Akt signaling pathway and TGF-*β* signaling pathway. Activation of PI3K-Akt signaling pathway may contribute to the development of Ewing's sarcoma [21, 22]. TGF-*β* signaling pathway could inhibit apoptosis and promote proliferation of Ewing's sarcoma cells [23]. Genes involved in the TGF-*β* signaling pathway might promote the progression of Ewing's sarcoma.

To identify common oncogenic genes both in osteosarcoma and Ewing's sarcoma, common DEGs both in osteosarcoma and Ewing's sarcoma were analyzed. A total of 201 genes were identified. Heatmap depicted that most of these genes had similar expression pattern both in osteosarcoma and Ewing's sarcoma cells compared to mesenchymal stem cells, indicating that these genes might be common oncogenic genes for osteosarcoma and Ewing's sarcoma. However, additional analysis needs to be performed. Afterwards, A PPI network based on these common genes was constructed. A hub gene plays a vital role in biological processes. In related pathways, the regulation of other genes is often dominated by this gene. 12 genes with degree > 10 were considered as hub genes. Intriguingly, these hub genes were all downregulated both in osteosarcoma and Ewing's sarcoma cells compared to mesenchymal stem cells. The 12 hub genes were as follows: FN1, COL1A2, COL1A1, POSTN, TIMP1, THBS1, SERPINE1, ITGA5, TIMP3, ADAMTS2, MMP13, and COMP. We further confirmed the expression patterns of these hub genes between osteosarcoma or Ewing's sarcoma tissues and normal tissues by RT-qPCR. Functional enrichment analysis results of common DEGs showed that these genes were mainly involved in ECM-receptor interaction (including FN1, COL1A1, COL1A2, COMP, ITGA5, and THBS1) and AGE-RAGE signaling pathway in diabetic complications (including FN1, COL1A1, COL1A2, SERPINE1, and AGTR1). We found that hub genes FN1, COL1A2, and COL1A1 were involved in the two pathways. Our western blot confirmed that FN1, COL1A2, and COL1A1 were reduced expression both in osteosarcoma and Ewing's sarcoma tissues compared to normal tissues. The composition and structure of ECM are known to be a key determinant of tumor metastasis. Recent studies have reported that activation of Wnt/*β*-catenin promotes the secretion of ECM proteins in tumor cells [24]. Consistent with the previous studies, in this study, ECM-receptor interaction was highly enriched by DEGs in osteosarcoma cells [25]. FN1 has been confirmed to be involved in ECM-receptor interaction [26]. It is upregulated in the chemo-resistant osteosarcoma cell lines and tissues and associated with poor prognosis [27, 28]. Our results showed that COL1A1 was downregulated in osteosarcoma cells, which were consistent with the previous study [29]. Furthermore, it has been reported that in the osteosarcoma cells, COL1A1 and FN1 could be associated with gastric cancer prognosis [30]. A previous study has found that COL1A2 and COL1A1 could be associated with TWIST1, a key transcription factor in metastasis [31]. Our GO enrichment analysis results showed that FN1, COL1A2, and COL1A1 were significantly enriched in the extracellular matrix, protease binding, and so on, indicating that the three genes could play a critical role in the development of osteosarcoma and Ewing's sarcoma.

In summary, observation of the common key genes in osteosarcoma and Ewing's sarcoma suggests that these specific genetic changes may be involved in regulation of the progression of osteosarcoma and Ewing's sarcoma. These hub genes can be used as candidate targets for the diagnosis and treatment of osteosarcoma and Ewing's sarcoma. Of course, there is more we can do. In our study, we only verified the effect of FN1, COL1A1, and COL1A2 genes on the drug sensitivity of cisplatin, and other first-line chemotherapeutics, such as methotrexate and Adriamycin, have not been studied.

## 4. Conclusion

To explore possible common oncogenic factors both in osteosarcoma and Ewing's sarcoma, we comprehensively analyzed the mRNA expression pattern in osteosarcoma cells and Ewing's sarcoma cells. Common key genes both in osteosarcoma and Ewing's sarcoma were identified, such as FN1, COL1A2, and COL1A1, which may act as antioncogene by enhancing cisplatin sensitivity in osteosarcoma cells and require further investigation.

## 5. Materials and Methods

### 5.1. Microarray Data

GSE70414 and GSE70826 microarray datasets were downloaded from the GEO (http://www.ncbi.nlm.nih.gov/geo/) database. GSE70414 dataset contains the mRNA expression data of five osteosarcoma cells and one human mesenchymal stem cell. GSE70826 microarray dataset contains the mRNA expression data of five Ewing's sarcoma cells and one human mesenchymal stem cell. The two datasets are based on the GPL570 platform. Expression levels of probes mapping into multiple genes were averaged.

### 5.2. Analysis of DEGs

According to the expression profiling data of GSE70414 and GSE70826, DEGs in osteosarcoma or Ewing's sarcoma cell lines compared with human mesenchymal stem cells were identified using the limma package (http://www.bioconductor.org/packages/release/bioc/html/limma.html). The genes with ∣log fold change (FC) | >1.5 and *p* < 0.05 were considered as DEGs. Unsupervised hierarchical clustering of different samples was performed using the R package based on microarray data.

### 5.3. Functional Enrichment Analysis

Functional enrichment analysis of DEGs was performed using the R language clusterProfiler package. Gene Ontology (GO) terms were significantly enriched by DEGs. GO analysis includes biological process, cellular component, and molecular function. The number of DEGs involved in GO terms was counted. Furthermore, we made Kyoto Encyclopedia of Genes and Genomes (KEGG) analysis, which was used to find the pathway terms involved in DEGs. *p* value < 0.05 was considered significantly enriched. The results were visualized using the Cytoscape plugin ClueGO. Gene set enrichment analysis (GSEA) was also performed, with the threshold of 1,000 permutations and a false discovery rate (FDR) < 0.25.

### 5.4. PPI Network Analysis

A PPI network was constructed by the STRING online database (http://string-db.org/) to predict the relationships among the products of differentially expressed genes [30]. The relationships of DEGs were visualized by use of Cytoscape (version 3.4.0) [31]. Nodes stand for biological molecules, and edges connected the nodes represent their interactions [14]. Furthermore, using the Cytoscape plugin MCODE, the most significant module was screened in the PPI network.

### 5.5. Patients and Specimens

38 osteosarcoma and 17 Ewing's sarcoma patients who underwent complete resection were recruited from the Shanghai Sixth People's Hospital between January 2014 and December 2015. The tumor tissue and the matched adjacent normal tissue were simultaneously collected from each patient. None of them experienced chemotherapy before surgery. This study gained the approval of the Ethical Committee of Shanghai Sixth People's Hospital, strictly following the Declaration of Helsinki (YS-2018-039). Each participant provided written informed consent. All the resection specimens were placed instantly into liquid nitrogen and stored at −80°C.  Table 1 lists the clinical characteristics of patients with osteosarcoma and Ewing's sarcoma. No significant differences in age, gender, and death were found between the two groups. But there was a significant difference in recurrence between the two groups (*p* = 0.0406).

Total RNA was extracted from tissues utilizing TRIzol (Invitrogen, Carlsbad, California, USA), which was reverse transcribed into cDNA via the reverse transcriptase kit (Invitrogen). PCR was presented with the TB Green® Premix Ex Taq™ II kit (TAKARA, Japan) according to the following procedures: 40 cycles of 94°C lasting 15 s, 60°C lasting 10 s, and 72°C lasting 20 s. Gene expression was normalized to GAPDH, followed by calculation of relative expression levels with the 2^−*ΔΔ*Ct^ method. The primer sequences are listed in Supplementary Table 8.

### 5.6. Cell Culture and Transfection

Human osteosarcoma cell line U2OS were purchased from Procell Life Science & Technology (Wuhan, China) and cultured in McCoy's 5A medium (Gibco, USA) containing 10% fetal bovine serum (Gibco) and 1% penicillin-streptomycin (Biosharp, China) at 37°C in a humidified atmosphere of 95% air and 5% CO2. U2OS cells were incubated with different concentrations of cisplatin (Sigma, Germany). The concentration was stepwisely increasing (0, 2 *μ*g/ml, 4 *μ*g/ml, 6 *μ*g/ml, 8 *μ*g/ml, and 10 *μ*g/ml) to calculate the cisplatin half maximal inhibitory concentration (IC50).

Small interfering RNA specific for FN1 (si-FN1), COL1A1 (si-COL1A1), COL1A2 (si-COL1A2), and negative control (NC) was purchased from Sangon Biotech (Shanghai, China). 100 nM of each item was transfected into U2OS cells using Lipofectamine 2000 (Invitrogen, USA) according to the manufacturer's instructions. The U2OS cells were harvested for further study after 48 h.

### 5.7. Cell Viability

Cell viability was determined by Cell Counting Kit-8 (CCK-8, Dojindo, Japan) assay. For the CCK-8 assay, U2OS cells with different treatments were cultured in 96-well plates at 5∗10^3^ cells per well. After the indicated times, exchange serum-free medium and add 10 *μ*l CCK-8 solution into each well. The absorbance at 450 nm was measured by a microplate reader (Molecular Devices, USA) after incubation at 37°C for 2 hours.

### 5.8. Flow Cytometry

An Annexin V-FITC/PI kit (BD Biosciences, USA) was used to determine the number of apoptotic cells according to the manufacturer's instructions. Briefly, U2OS cells were harvested and washed twice with cold PBS and resuspended in 300 *μ*l of binding buffer. The cell samples were incubated with 10 *μ*l Annexin V-allophycocyanin (FITC) solution and 5 *μ*l propidium iodide (PI) solution for 15 minutes in the dark at room temperature. The ratio of apoptotic cells was measured by FACS Calibur (BD Biosciences).

### 5.9. Transwell Assay

A total of 2∗10^4^ U2OS cells in serum-free medium were seeded into the upper chamber, while the lower chamber was maintained in 10% FBS medium. After incubation for 24 h at room temperature, migratory cells at the bottom of the upper chamber were fixed with 4% paraformaldehyde for 30 min, stained with crystal violet for 10 min, and then counted under an inverted microscope (Leica, Germany).

### 5.10. RT-PCR

The transcription level of osteogenic genes was detected by reverse transcription PCR (RT-PCR). The reaction system used was SYBR Green Mix (Takara, RR420A), and the fluorescence signal was obtained by a detecting instrument (Roche, Light Cycler 480).

### 5.11. Statistical Analysis

Statistical analysis was carried out via R 3.6.3 and GraphPad 7.0. Data from experiments are expressed as means ± standard deviation. Differences in clinical features between osteosarcoma and Ewing's sarcoma groups were assessed by chi-square test. Paired Student's *t* test was used for comparisons between the two groups. Differences with *p* < 0.05 was considered statistically significant.

## Figures and Tables

**Table 1:** The clinical characteristics of patients with osteosarcoma and Ewing's sarcoma.

Parameters	Osteosarcoma (*N* = 38)	Ewing's sarcoma (*N* = 17)	*p* value
Age (year)			0.3711
≤18	27	10	
>18	11	7	
Gender			0.9378
Male	25	11	
Female	13	6	
Recurrence			0.0406
Yes	6	7	
No	32	10	
Death			0.0550
Yes	10	9	
No	28	8	

## Data Availability

The datasets used and/or analyzed during the current study are available from the corresponding author on reasonable request.

## Abbreviations

DEGs: Differentially expressed genes
GEO: Gene Expression Omnibus
FC: Fold change
GO: Gene Ontology
KEGG: Kyoto Encyclopedia of Genes, and Genomes
PPI: Protein-protein interaction
RT-PCR: Reverse transcription-polymerase chain reaction
GSEA: Gene set enrichment analysis
FDR: False discovery rate.

## Supplementary Materials

Supplementary 1 Supplementary Figure 1: common DEGs both in osteosarcoma and Ewing's sarcoma. (A) Venn diagram showing 201 common DEGs both in osteosarcoma and Ewing's sarcoma. Heatmap showing the difference in expression pattern of these common DEGs between osteosarcoma cells (B) and Ewing's sarcoma cells (C). Red stands for upregulated genes and green stands for downregulated genes.

Supplementary 2 Supplementary Figure 2: a PPI network of 201 common DEGs both in osteosarcoma and Ewing's sarcoma. (A) A PPI network. (B) A subnetwork. Red suggests upregulated genes and blue suggests downregulated genes.

Supplementary 3 Supplementary Figure 3: biological processes of common DEGs both in osteosarcoma and Ewing's sarcoma compared to mesenchymal stem cell.

Supplementary 4 Supplementary Figure 4: cell component results of common DEGs both in osteosarcoma and Ewing's sarcoma compared to mesenchymal stem cell.

Supplementary 5 Supplementary Figure 5: molecular function results of common DEGs both in osteosarcoma and Ewing's sarcoma compared to mesenchymal stem cell.

Supplementary 6 Supplementary Figure 6: KEGG pathway results of common DEGs both in osteosarcoma and Ewing's sarcoma compared to mesenchymal stem cell.

Supplementary 7 Supplementary Table 1: the top ten upregulated genes in osteosarcoma cells compared to mesenchymal stem cells.

Supplementary 8 Supplementary Table 2: the top ten downregulated genes in osteosarcoma cells compared to mesenchymal stem cells.

Supplementary 9 Supplementary Table 3: KEGG pathways and enriched genes for osteosarcoma cells.

Supplementary 10 Supplementary Table 4: the top ten upregulated genes in Ewing's sarcoma cells compared to mesenchymal stem cells.

Supplementary 11 Supplementary Table 5: the top ten downregulated genes in Ewing's sarcoma cells compared to mesenchymal stem cells.

Supplementary 12 Supplementary Table 6: KEGG pathways and enriched genes associated with Ewing's sarcoma.

Supplementary 13 Supplementary Table 7: the genes with degree > 10 in the PPI network.

Supplementary 14 Supplementary Table 8: primer sequences for real-time PCR.
