# Supplementary figures and images for: Identification of Common Oncogenic Genes and Pathways Both in Osteosarcoma and Ewing's Sarcoma Using Bioinformatics Analysis
Source: J Immunol Res. 2022 May 5;2022:3655908. doi: 10.1155/2022/3655908 (PMC9107040; doi:10.1155/2022/3655908)

A

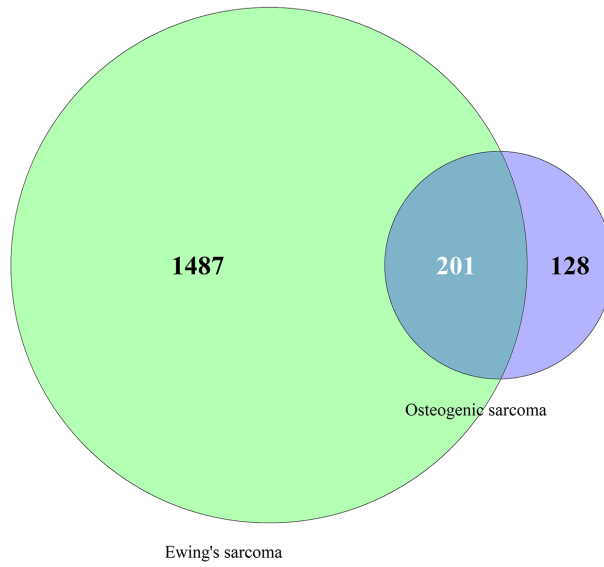

B

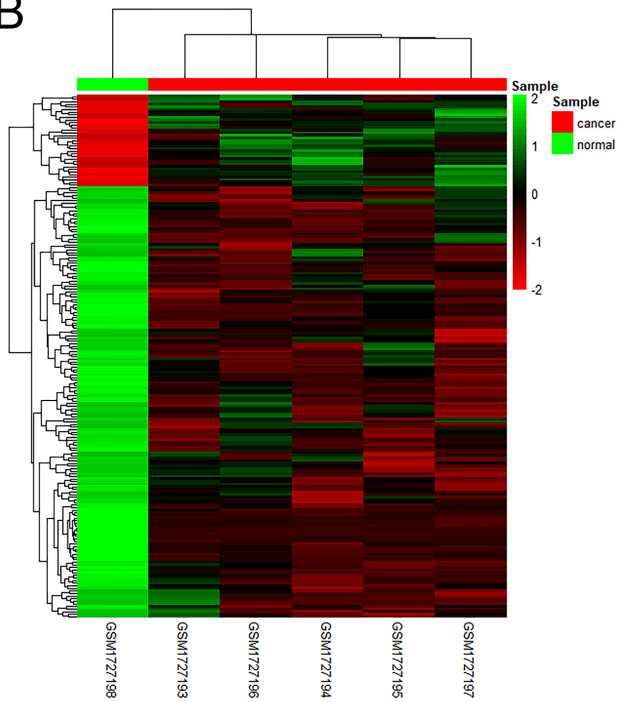

C

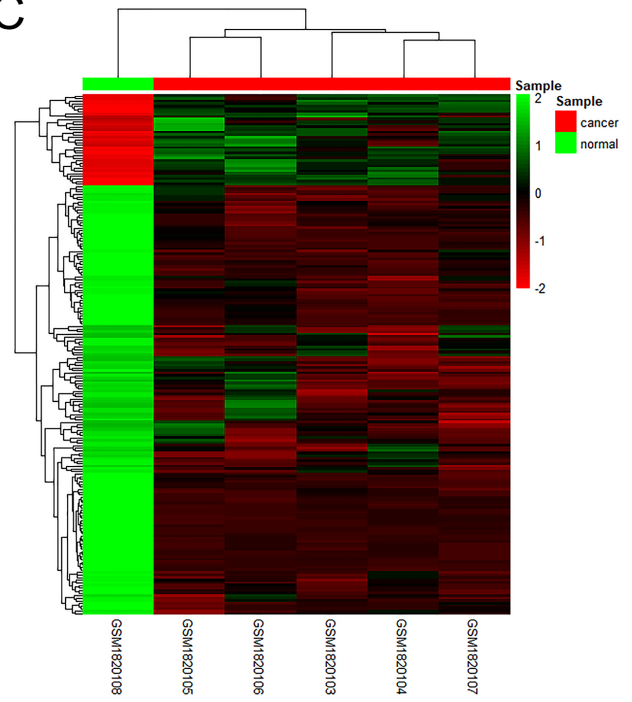

Supplement: Supplementary 1 — Supplementary Figure 1: common DEGs both in osteosarcoma and Ewing's sarcoma. (A) Venn diagram showing 201 common DEGs both in osteosarcoma and Ewing's sarcoma. Heatmap showing the difference in expression pattern of these common DEGs between osteosarcoma cells (B) and Ewing's sarcoma cells (C). Red stands for upregulated genes and green stands for downregulated genes. [file 3655908.f1.pdf]

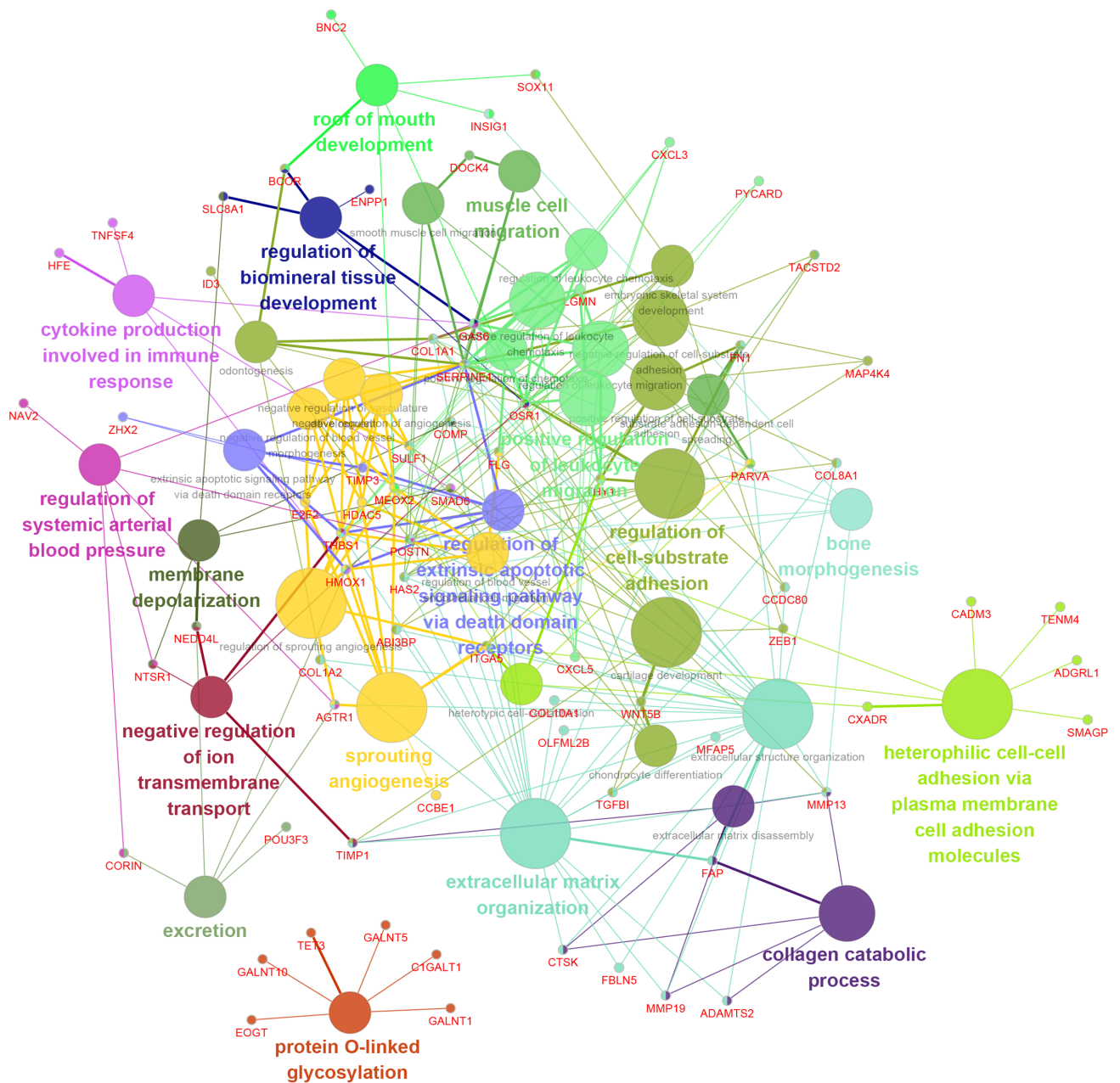

Supplement: Supplementary 3 — Supplementary Figure 3: biological processes of common DEGs both in osteosarcoma and Ewing's sarcoma compared to mesenchymal stem cell. [file 3655908.f3.pdf]

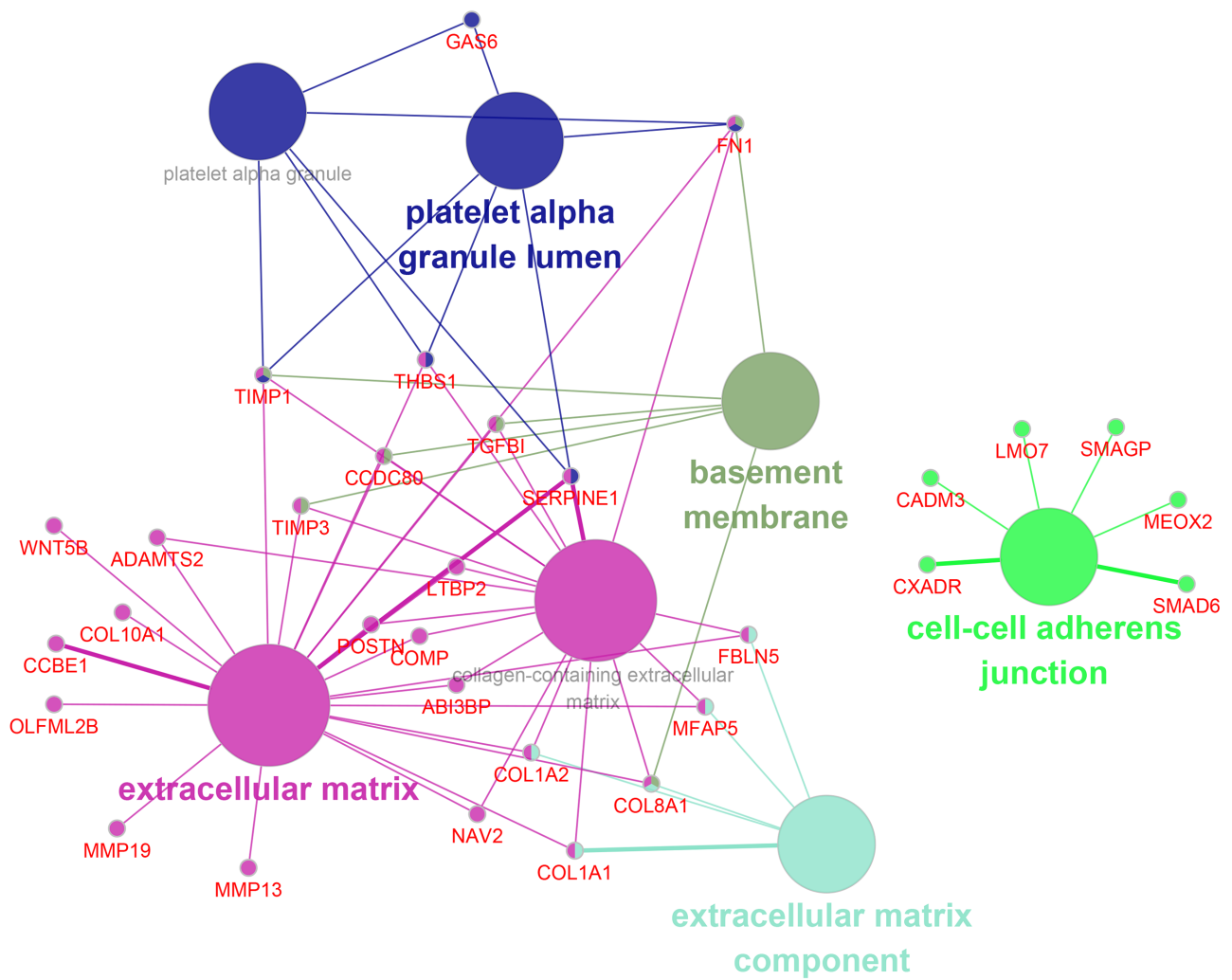

Supplement: Supplementary 4 — Supplementary Figure 4: cell component results of common DEGs both in osteosarcoma and Ewing's sarcoma compared to mesenchymal stem cell. [file 3655908.f4.pdf]

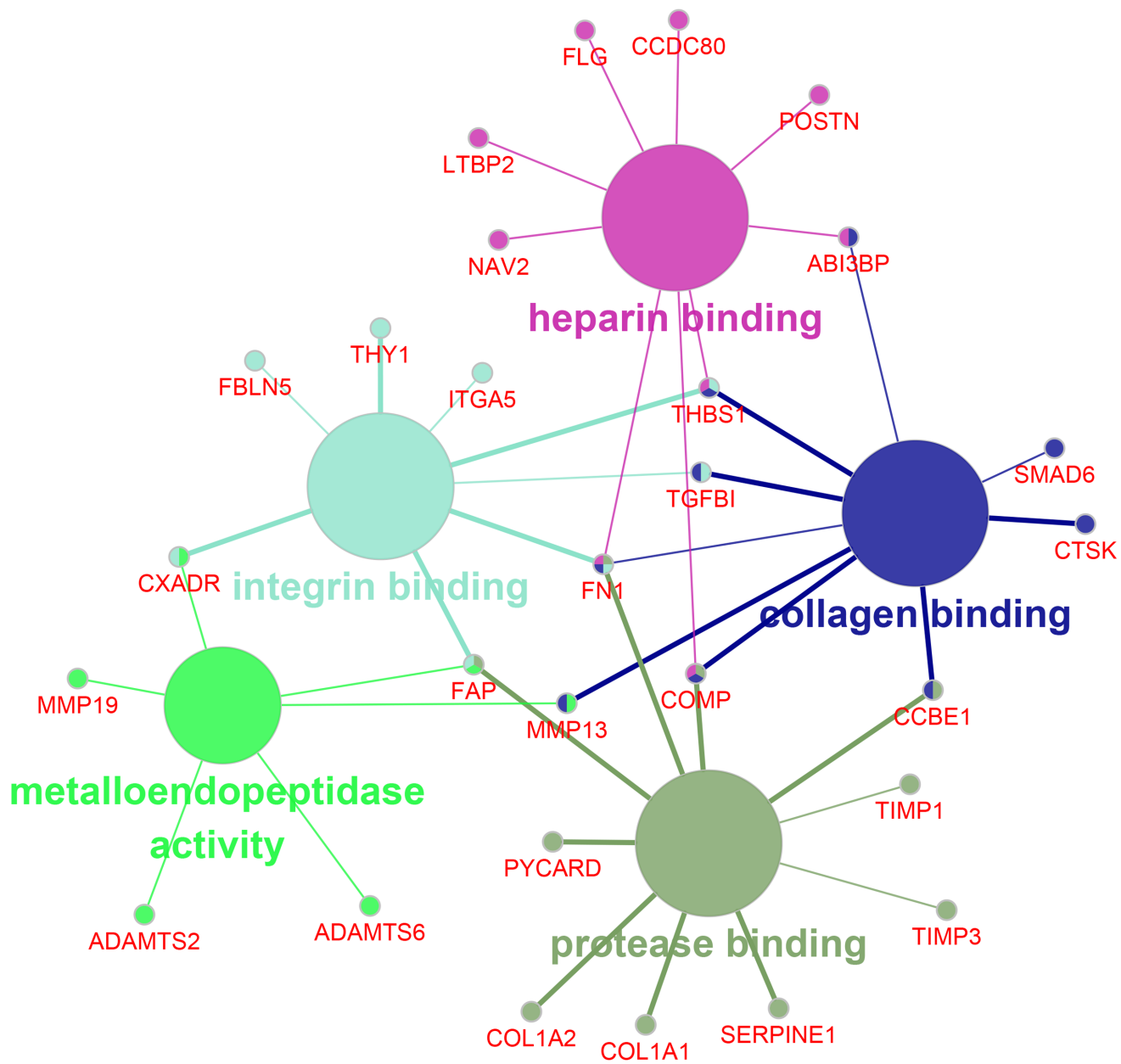

Supplement: Supplementary 5 — Supplementary Figure 5: molecular function results of common DEGs both in osteosarcoma and Ewing's sarcoma compared to mesenchymal stem cell. [file 3655908.f5.pdf]

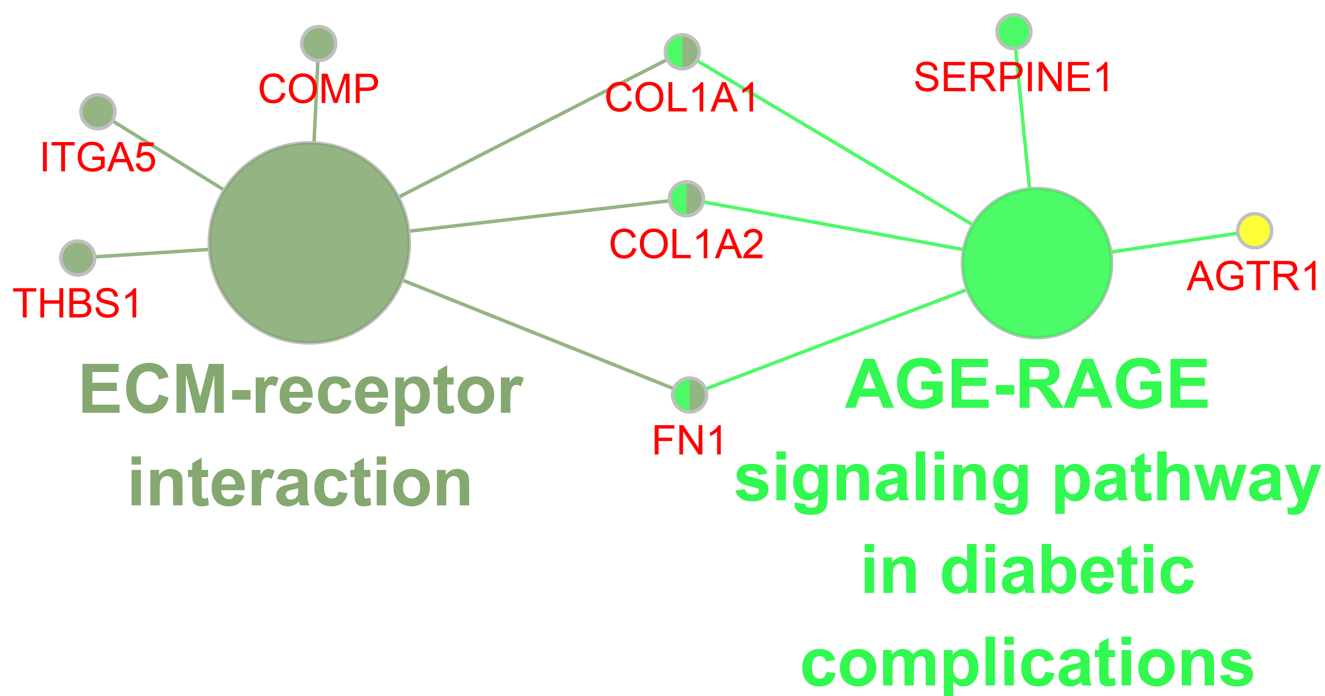

Supplement: Supplementary 6 — Supplementary Figure 6: KEGG pathway results of common DEGs both in osteosarcoma and Ewing's sarcoma compared to mesenchymal stem cell. [file 3655908.f6.pdf]
