# Supplementary material for: Identification of Common Oncogenic Genes and Pathways Both in Osteosarcoma and Ewing's Sarcoma Using Bioinformatics Analysis
Source: J Immunol Res. 2022 May 5;2022:3655908. doi: 10.1155/2022/3655908 (PMC9107040; doi:10.1155/2022/3655908)
Supplement: Supplementary 2 — Supplementary Figure 2: a PPI network of 201 common DEGs both in osteosarcoma and Ewing's sarcoma. (A) A PPI network. (B) A subnetwork. Red suggests upregulated genes and blue suggests downregulated genes. [file 3655908.f2.pdf]

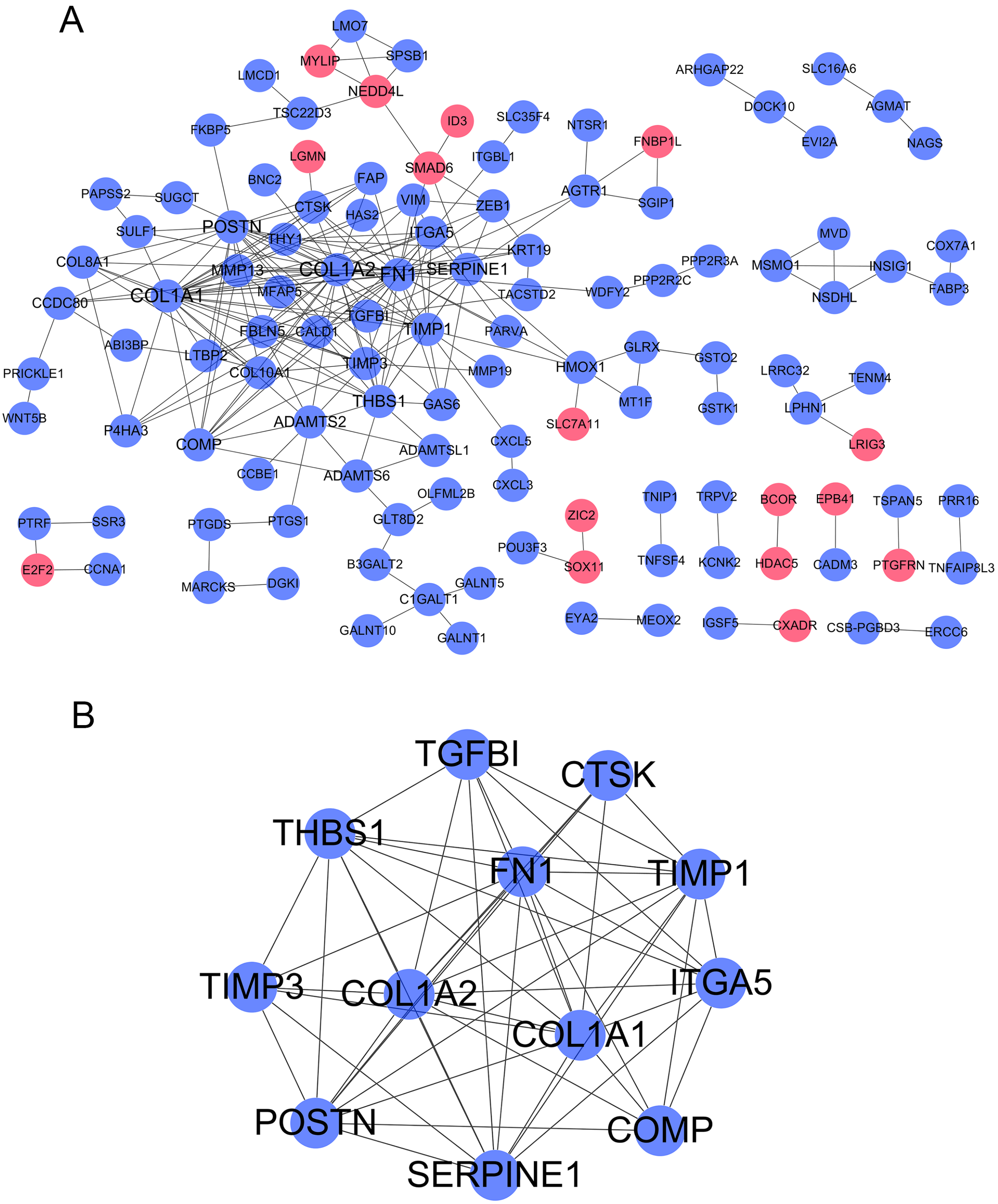

A
LMO7
MYLIP
SPSB1
LMCD1
NEDD4L
TSC22D3
FKBP5
ID3
SLC35F4
NTSR1
FNBP1L
LGMN
SMAD6
ITGBL1
BNC2
FAP
PAPSS2
SUGCT
CTSK
VIM
ZEB1
AGTR1
SGIP1
HAS2
SULF1
POSTN
THY1
ITGA5
KRT19
COL8A1
MMP13
COL1A2
FN1
SERPINE1
CCDC80
COL1A1
MFAP5
TACSTD2
WDFY2
PPP2R2C
PPP2R3A
TGFBI
FBLN5
CALD1
TIMP1
PARVA
ABI3BP
LTBP2
TIMP3
MMP19
HMOX1
GLRX
GSTO2
PRICKLE1
COL10A1
MT1F
THBS1
GAS6
GSTK1
WNT5B
P4HA3
SLC7A11
COMP
ADAMTS2
ADAMTSL1
CXCL5
CCBE1
ADAMTS6
CXCL3
OLFML2B
ARHGAP22
DOCK10
EVI2A
SLC16A6
AGMAT
NAGS
MVD
COX7A1
MSMO1
INSIG1
FABP3
NSDHL
LRRC32
TENM4
LPHN1
LRIG3
PTRF
SSR3
E2F2
CCNA1
PTGDS
PTGS1
MARCKS
DGKI
GLT8D2
B3GALT2
GALNT5
C1GALT1
GALNT10
GALNT1
ZIC2
POU3F3
SOX11
TNIP1
TNFSF4
TRPV2
KCNK2
BCOR
HDAC5
EPB41
CADM3
TSPAN5
PTGFRN
PRR16
TNFAIP8L3
EYA2
MEOX2
IGSF5
CXADR
CSB-PGBD3
ERCC6
B
TGFBI
CTSK
THBS1
FN1
TIMP1
TIMP3
COL1A2
ITGA5
COL1A1
POSTN
COMP
SERPINE1
