# Supplementary material for: Identification of Common Oncogenic Genes and Pathways Both in Osteosarcoma and Ewing's Sarcoma Using Bioinformatics Analysis
Source: J Immunol Res. 2022 May 5;2022:3655908. doi: 10.1155/2022/3655908 (PMC9107040; doi:10.1155/2022/3655908)
Supplement: Supplementary 7 — Supplementary Table 1: the top ten upregulated genes in osteosarcoma cells compared to mesenchymal stem cells. [file 3655908.f7.pdf]

Supplementary Table 1. The top ten up-regulated genes in osteosarcoma cells compared to mesenchymal stem cells.

Gene symbol	Gene title	P-value	logFC
LOC728613	programmed cell death 6 pseudogene	0.015023	5.62348
ZIC2	Zic family member 2	0.004358	5.219626
CD24	CD24 molecule	0.021227	5.059026
ABLIM1	actin binding LIM protein 1	0.000458	5.011336
MYLIP	myosin regulatory light chain interacting protein	0.0022	4.020656
SIPA1L2	signal induced proliferation associated 1 like 2	0.000255	3.943124
RHPN2	rhophilin Rho GTPase binding protein 2	0.014097	3.92881
S100A4	S100 calcium binding protein A4	0.032193	3.62968
LHX2	LIM homeobox 2	0.002175	3.577594
LOC100996740	uncharacterized LOC100996740	0.031393	3.551738
