# Supplementary material for: Identification of Common Oncogenic Genes and Pathways Both in Osteosarcoma and Ewing's Sarcoma Using Bioinformatics Analysis
Source: J Immunol Res. 2022 May 5;2022:3655908. doi: 10.1155/2022/3655908 (PMC9107040; doi:10.1155/2022/3655908)
Supplement: Supplementary 8 — Supplementary Table 2: the top ten downregulated genes in osteosarcoma cells compared to mesenchymal stem cells. [file 3655908.f8.pdf]

Supplementary Table 2. The top ten down-regulated genes in osteosarcoma cells compared to mesenchymal stem cells.

Gene symbol	Gene title	P-value	logFC
FBLN5	fibulin 5	0.001291	-7.41923
PLD5	phospholipase D family member 5	1.91E-07	-5.80124
TRIM22	tripartite motif containing 22	8.60E-07	-5.65307
FN1	fibronectin 1	2.26E-06	-5.52538
KCTD12	potassium channel tetramerization domain containing 12	0.010454	-5.50432
CTSK	cathepsin K	0.001203	-5.46193
ABI3BP	ABI family member 3 binding protein	3.44E-07	-5.12252
HAS2	hyaluronan synthase 2	0.038316	-5.02986
POSTN	periostin	2.83E-07	-5.01706
TACSTD2	tumor-associated calcium signal transducer 2	2.07E-07	-4.97786
