# Supplementary material for: Identification of Common Oncogenic Genes and Pathways Both in Osteosarcoma and Ewing's Sarcoma Using Bioinformatics Analysis
Source: J Immunol Res. 2022 May 5;2022:3655908. doi: 10.1155/2022/3655908 (PMC9107040; doi:10.1155/2022/3655908)
Supplement: Supplementary 9 — Supplementary Table 3: KEGG pathways and enriched genes for osteosarcoma cells. [file 3655908.f9.pdf]

Supplementary Table 3. KEGG pathways and enriched genes for osteosarcoma cells.

ID	Description	Gene symbol
hsa04512	ECM-receptor interaction	COL1A1/COL1A2/COMP/FN1/ITGA4/ITGA5/THBS1
hsa04933	AGE-RAGE signaling pathway in diabetic complications	COL1A1/COL1A2/AGTR1/FN1/SMAD3/NOX4/SERPINE1
hsa00512	Mucin type O-glycan biosynthesis	GALNT5/GALNT1/GALNT10/C1GALT1
hsa04510	Focal adhesion	COL1A1/COL1A2/COMP/FN1/ITGA4/ITGA5/PAK1/PARVA/RAC2/THBS1
hsa00100	Steroid biosynthesis	DHCR7/NSDHL/MSMO1
hsa05206	MicroRNAs in cancer	BCL2L11/E2F2/FOXP1/HMOX1/ITGA5/MIR34A/MARCKS/ZEB1/THBS1/TIMP3/VIM/RPS6KA5
hsa04115	p53 signaling pathway	DDB2/IGFBP3/SERPINE1/THBS1/TP53I3
