# Supplementary material for: Identification of Common Oncogenic Genes and Pathways Both in Osteosarcoma and Ewing's Sarcoma Using Bioinformatics Analysis
Source: J Immunol Res. 2022 May 5;2022:3655908. doi: 10.1155/2022/3655908 (PMC9107040; doi:10.1155/2022/3655908)
Supplement: Supplementary 11 — Supplementary Table 5: the top ten downregulated genes in Ewing's sarcoma cells compared to mesenchymal stem cells. [file 3655908.f11.pdf]

Supplementary Table 5. The top ten down-regulated genes in Ewing's sarcoma cells compared to mesenchymal stem cells.

Gene symbol	Gene title	P-value	logFC
COL6A3	collagen type VI alpha 3 chain	4.88E-06	-9.17661
COL8A1	collagen type VIII alpha 1 chain	2.17E-08	-8.68956
CTHRC1	collagen triple helix repeat containing 1	0.000212	-8.6274
SRGN	serglycin	0.000297	-8.28863
TGFBI	transforming growth factor beta induced	1.41E-06	-8.10883
MICAL2	microtubule associated monooxygenase, calponin and LIM domain containing 2	2.17E-06	-8.01617
ITGBL1	integrin subunit beta like 1	6.60E-08	-7.94664
HAS2	hyaluronan synthase 2	6.38E-08	-7.80376
LGALS3	lectin, galactoside binding soluble 3	1.70E-05	-7.76557
SERPINE1	serpin family E member 1	3.85E-06	-7.37805
