# Supplementary material for: Identification of Common Oncogenic Genes and Pathways Both in Osteosarcoma and Ewing's Sarcoma Using Bioinformatics Analysis
Source: J Immunol Res. 2022 May 5;2022:3655908. doi: 10.1155/2022/3655908 (PMC9107040; doi:10.1155/2022/3655908)
Supplement: Supplementary 12 — Supplementary Table 6: KEGG pathways and enriched genes associated with Ewing's sarcoma. [file 3655908.f12.pdf]

**Supplementary Table 6. KEGG pathways and enriched genes associated with Ewing's sarcoma.**

| Description | Gene symbol |
|---|---|
| Focal adhesion | MYL9/MYL12A/COL1A1/COL1A2/COL4A1/COL6A1/COL6A2/COL6A3/COL9A3/COMP/EGFR/ITGA11/FN1/LAMA1/PARVB/HGF/TNC/ITGA3/ITGA5/ITGB3/ITGB5/LAMC1/MET/PDGFA/PDGFRA/PDGFRB/PIK3CD/PARVA/PRKCA/PRKCB/PDGFC/PTEN/PTK2/THBS1/THBS2/VASP/VEGFA/VEGFC/ZYX/ITGA10/ACTN1/CCND2 |
| ECM-receptor interaction | COL1A1/COL1A2/COL4A1/COL6A1/COL6A2/COL6A3/COL9A3/COMP/ITGA11/FN1/LAMA1/TNC/ITGA3/ITGA5/ITGB3/ITGB5/LAMC1/SDC1/SDC4/THBS1/THBS2/ITGA10/CD44/CD47 |
| Human papillomavirus infection | CDK6/TCIRG1/IRF9/COL1A1/COL1A2/COL4A1/COL6A1/COL6A2/COL6A3/COL9A3/COMP/DLG2/EGFR/ITGA11/FN1/HEY1/ATP6V0A2/LAMA1/HLA-E/HES1/TNC/RBPJ/ITGA3/ITGA5/ITGB3/ITGB5/JAK1/LAMC1/NOTCH1/NOTCH2/PDGFRB/PIK3CD/PPP2R2A/PPP2R2C/PPP2R3A/HES6/PTEN/PTGER4/PTK2/THBS1/THBS2/TP53/TRAF3/VEGFA/FZD3/WNT5B/FZD6/FZD7/TCF7L1/PARD6B/ITGA10/CCNA1/CCND2/ATP6V1E2/CREB3L1/ATP6V0D1 |
| PI3K-Akt signaling pathway | CDK6/THEM4/COL1A1/COL1A2/COL4A1/COL6A1/COL6A2/COL6A3/COL9A3/COMP/LPAR1/EFNA1/EFNA4/EGFR/FGF1/FGF5/ITGA11/FN1/GNG4/LAMA1/HGF/TNC/IL4R/INSR/ITGA3/ITGA5/ITGB3/ITGB5/JAK1/AREG/KIT/LAMC1/MET/MYB/MYC/PDGFA/PDGFRA/PDGFRB/PIK3CD/PPP2R2A/PPP2R2C/PPP2R3A/PRKCA/GNG12/PDGFC/PTEN/PTK2/BCL2L1/THBS1/THBS2/TP53/VEGFA/VEGFC/ITGA10/CCND2/CREB3L1/LPAR2/OSMR |
| Proteoglycans in cancer | TWIST2/CTSL/DDX5/EGFR/FN1/GPC1/HGF/HIF1A/HOXD10/ITGA5/ITGB3/ITGB5/LUM/MET/MMP2/MYC/PLCE1/PIK3CD/PLAU/PLAUR/PRKCA/PRKCB/PTCH1/PTK2/SDC1/SDC4/SLC9A1/THBS1/TIMP3/TP53/VEGFA/FZD3/WNT5B/CAMK2D/FZD6/FZD7/IQGAP1/CD44/CD63 |
| Mineral absorption | SLC40A1/HMOX1/MT1E/MT1F/MT1G/MT1H/MT1M/MT1X/MT2A/ATP1A1/MT1HL1/SLC8A1/VDR/CYBRD1/HEPH |
| TGF-beta signaling pathway | CDKN2B/FST/E2F5/BAMBI/RGMB/ID2/ID3/ID4/INHBA/LTBP1/SMAD6/SMAD9/MYC/NBL1/TGIF2/SMURF2/BMP4/THBS1/ACVR2A/ACVR2B |
| Lysosome | TCIRG1/NPC2/AP3M2/CTSB/CTSK/CTSL/CTSO/CTSZ/GGA2/ATP6V0A2/PLA2G15/FUCA1/SLC17A5/SUMF1/LAMP2/LIPA/MANBA/NEU1/NPC1/LAPTM4B/LGMN/SORT1/ATP6V0D1/CD63 |
| Arrhythmogenic right ventricular cardiomyopathy (ARVC) | CDH2/DSP/ITGA11/ITGA3/ITGA5/ITGB3/ITGB5/JUP/LMNA/PKP2/RYR2/SGCB/SGCD/SLC8A1/CACNB2/TCF7L1/ITGA10 |
| Fluid shear stress and | GSTO2/CTSL/CYBA/NQO1/DUSP1/GPC1/GSTM4/GSTT1/GSTT2/HMOX1/IL1R1/ITGB3/MMP2/PDGFA/PIK3CD/PLAT/MAP2K5/M |

| | |
|---|---|
| atherosclerosis | AP2K6/PTK2/SDC1/SDC4/BMP4/TP53/VEGFA/ACVR2A/ACVR2B |
