# Supplementary material for: Identification of Common Oncogenic Genes and Pathways Both in Osteosarcoma and Ewing's Sarcoma Using Bioinformatics Analysis
Source: J Immunol Res. 2022 May 5;2022:3655908. doi: 10.1155/2022/3655908 (PMC9107040; doi:10.1155/2022/3655908)
Supplement: Supplementary 13 — Supplementary Table 7: the genes with degree > 10 in the PPI network. [file 3655908.f13.pdf]

Supplementary Table 7. The genes with degree>10 in the PPI network.

Name	Degree	Betweenness centrality	Closeness centrality	Clustering coefficient	Stress	Average shortest path length
FN1	28	0.311	0.446	0.238	6142	2.241
COL1A2	27	0.162	0.427	0.262	4216	2.342
COL1A1	26	0.121	0.425	0.277	3566	2.354
POSTN	19	0.100	0.397	0.363	2458	2.519
TIMP1	16	0.098	0.397	0.433	2212	2.519
THBS1	15	0.124	0.407	0.486	3204	2.456
SERPINE1	15	0.101	0.407	0.457	2902	2.456
ITGA5	12	0.058	0.373	0.500	1084	2.684
TIMP3	12	0.026	0.376	0.530	1006	2.658
ADAMTS2	10	0.142	0.351	0.333	4292	2.848
MMP13	10	0.003	0.367	0.756	168	2.722
COMP	10	0.074	0.393	0.556	2296	2.544
