# Supplementary material for: Identification of Common Oncogenic Genes and Pathways Both in Osteosarcoma and Ewing's Sarcoma Using Bioinformatics Analysis
Source: J Immunol Res. 2022 May 5;2022:3655908. doi: 10.1155/2022/3655908 (PMC9107040; doi:10.1155/2022/3655908)
Supplement: Supplementary 14 — Supplementary Table 8: primer sequences for real-time PCR. [file 3655908.f14.pdf]

Supplementary Table 8. Primer sequences for Real time PCR.

mRNAs	5'-3' sequences
FN1-F	GGGGTCACCTATTACTTCA
FN1-R	CTCTTGGTTGCCCTTTAT
COL1A2-F	TGCCTAGCAACATGCCAATC
COL1A2-R	GCAAAGTTCCCACCGAGA
COL1A1-F	TTTGAAGCCCAAGATGTAGAAG
COL1A1-R	CAGTGCTCAGCATCGGTCA
POSTN-F	GGGCAAATACTGGAAACC
POSTN-R	AGGCTGAGGAAGGTGCTA
TIMP1-F	TTCCACAGGTCCCACAAC
TIMP1-R	GCATTCCTCACAGCCAAC
THBS1-F	GACAGCATCCGCAAAGTG
THBS1-R	TATCAACAGTCCATTCCTCGT
SERPINE1-F	CTTTGGTGAAGGGTCTGC
SERPINE1-R	GGGTTTCTCCTCCTGTTGT
ITGA5-F	TCAGGAGCAGATTGCAGAA
ITGA5-R	GACATAGCCGTAAGTGAGGTT
TIMP3-F	CTGGCTACCAGTCCAAACAC
TIMP3-R	GGGAAGGGAGGGAAGTGA
ADAMTS2-F	CCAGCAGAAGCCAGACACG
ADAMTS2-R	TCTCATGGGCCACCACAAA
β-actin-F	CACAGAGCCTCGCCTTTGCC
β-actin-R	ACCCATGCCCACCATCACG
